# Ethyl methane sulfonate induced mutations in M_2_ generation and physiological variations in M_1_ generation of peppers (*Capsicum annuum* L.)

**DOI:** 10.3389/fpls.2015.00399

**Published:** 2015-06-04

**Authors:** Mohamed H. Arisha, Syed N. M. Shah, Zhen-Hui Gong, Hua Jing, Chao Li, Huai-Xia Zhang

**Affiliations:** ^1^College of Horticulture, Northwest A&F University, YanglingChina; ^2^State Key Laboratories for Stress Biology of Arid Region Crop, Northwest A&F University, YanglingChina; ^3^Department of Horticulture, Faculty of Agriculture, Zagazig University, ZagazigEgypt; ^4^Department of Horticulture, Faculty of Agriculture, Gomal University, Dera Ismail KhanPakistan

**Keywords:** pepper, *Capsicum annuum*, EMS, mutations, M_1_ generation, M_2_ generation

## Abstract

This study was conducted to enhance genetic variability in peppers (*Capsicum annuum*, cv B12) using ethyl methanesulphonate (EMS). Exposure to an EMS concentration of 0.6%, v/v for 12 h was used to mutagenize 2000 seeds for the first generation (M_1_). It was observed that the growth behaviors including plant height, flowering date, and number of seeds per first fruit were different in the M_1_ generation than in wild type (WT) plants. In addition one phenotypic mutation (leaf shape and plant architecture) was observed during the M_1_ generation. During the seedling stage in the M_2_ generation, the observed changes were in the form of slow growth or chlorophyll defect (e.g., albino, pale green, and yellow seedlings). At maturity, there were three kinds of phenotypic mutations observed in three different families of the mutant population. The first observed change was a plant with yellow leaf color, and the leaves of this mutant plant contained 62.19% less chlorophyll a and 64.06% less chlorophyll b as compared to the wild-type. The second mutation resulted in one dwarf plant with a very short stature (6 cm), compact internodes and the leaves and stem were rough and thick. The third type of mutation occurred in four plants and resulted in the leaves of these plants being very thick and longer than those of WT plants. Furthermore, anatomical observations of the leaf blade section of this mutant plant type contained more xylem and collenchyma tissue in the leaf midrib of the mutant plant than WT. In addition, its leaf blade contained thicker palisade and spongy tissue than the WT.

## Introduction

Peppers (*Capsicum annuum* L.) are one of the most cultivated vegetable crops in tropical and subtropical regions. Inducing mutations in peppers is important for pepper breeding and crop improvement (Bosland and Votava, 2012). EMS, as a chemical mutagen, can be used as a supplementary approach to improve desired identifiable characters such as yield related characters (Botticella et al., 2011). It produces random point mutations in genetic material. Mutation frequency, detected using various techniques, displays a wide range of variation according the EMS treatment conditions. Mutation frequency per gene differs from one crop to another starting from one mutation per Mb in barley (Caldwell et al., 2004), to one mutation per 170 kb in *Arabidopsis* (Greene et al., 2003), one mutation per 50 kb in pepper (Pathirana, 2012; Just et al., 2013), and one mutation per 25 kb in the polyploid (hexaploid) wheat (Slade et al., 2004). Lethal dose 50 (LD_50_) can be defined as the EMS concentration and conditions that contributes to 50% lethality (out of the total number of seeds). Determination of LD_50_ is necessary to produce a high frequency of desirable mutations (Hohmann et al., 2005; Arisha et al., 2014).

In the M_1_ generation, only mutations of dominant characters can be identified, and it is not possible to identify mutations in a recessive character. Moreover, in the M_1_ generation, there are some signals for mutagen efficiency, for example: pollen sterility, reduction in plant height, late or early flowering, or curled leaves (Arisha et al., 2014). In the M_2_ generation, the mutation will segregate to create homozygotes for recessive or dominant alleles (Page and Grossniklaus, 2002). At that time visual screening is the most effective way to identify the phenotypic mutation. Visual screening can be used as a primary indicator to select plants that have desired characters, for example: disease resistance, flowering earliness, plant height, fruit color changes, or growth period (Østergaard and Yanofsky, 2004).

Presence of chlorophyll mutations in the mutant population is the most dependable index for evaluating the genetic effects of mutagenic treatments. In addition, chlorophyll mutations are important for identifying gene function and elucidation of chlorophyll metabolism and its regulation (Wu et al., 2007). Chlorophyll development seems to be controlled by many genes located on several chromosomes, which could be adjacent to centromeres or on proximal segments of chromosomes (Swaminathan, 1964). Another possible use for mutants is to detect dwarf mutants (Daskalov, 1986), which have been previously identified in peppers (Lippert et al., 1964; Alcantara et al., 1996; Jabeen and Mirza, 2004). In addition, induction of mutations affecting pepper plant architecture and leaf shape can provide us with information about the genetic control of plant architecture, since these characters have only been studied in detail in a limited number of model species such as *Arabidopsis* (Angenent et al., 2005), *Lycopersicon esculentum* (Prusinkiewicz et al., 2007), and *Petunia hybrida* (Quinet and Kinet, 2007).

Induced mutations can rapidly create variability in quantitatively and qualitatively inherited traits in crops. Therefore, the objective of this study is to obtain new germplasm to use in the improvement of the B12 pepper cultivar.

## Materials and Methods

### Plant Material

B12, a sweet pepper cultivar (*C. annuum* L.), was used in this experiment. Pepper seeds were provided by the *Capsicum* Research Group from the College of Horticulture at Northwest A&F University, China.

#### Experimental Procedures

In our previous study we standardized the EMS treatment in (*C. annuum* cv B12) by conducting a kill curve analysis (Arisha et al., 2014). Therefore, the concentration of 0.6%, V/V EMS for 12 h was chosen to mutagenize 2000 seeds. EMS solution was prepared in a 0.1 M phosphate buffer at pH 7.0 to avoid rapid hydrolysis (Bosland, 2002).

Seeds (2000) were presoaked in water for 6 h then treated with the above-mentioned concentration of EMS at 20°C with orbital shaking (110 rpm), while 50 seeds were used as a control (untreated). Seeds were then thoroughly washed under running water for 3 h and transferred to petri dishes containing wet filter paper and kept in a growth chamber at 28°C in the dark until germination (at 10 days after the treatment). Control seeds (untreated) were exposed to the same conditions except for the EMS treatment.

All germinated and non-germinated seeds were sown into pots that were placed in a greenhouse and standard cultural practices were carried-out thereafter. When four to six true leaves had grown, seedlings were transferred to soil under high tunnels to grow until maturity. Phenotypic data during the M_1_ generation were recorded. At the fruit ripening stage, the mature seeds of the first fruit from each individual was collected, packed, labeled, air dried, and stored in a freezer at -20°C (Arisha et al., 2014).

Thirty seeds from each M_1_ individual were germinated in petri dishes at 28°C. All germinated and non-germinated seeds were sown in one pot and the germination ratio was recorded. M_2_ seedlings with four to six true leaves were transferred to soil under high tunnels and grow there to maturity stage. The M_2_ population was screened for morphological mutants (from germination until maturity) and the survival percentage was recorded.

#### Parameters Studied

##### M_1_ generation

During the M_1_ generation, the germination percentage at 7 days after germination and the survival percentage were recorded (at the age of four to six true leaves). Moreover, a comparison between mutant and wild type (WT) populations for growth parameters [e.g., plant height (cm) at 100 DAS, days to flowering and number of seeds/first fruit) was conducted. Growth abnormalities were observed and recorded for the M_1_ generation compared to the WT plants.

##### M_2_ generation

Germination percentage at 7 days after starting germination and survival ratio (at maturity) for each family of M_2_ population was recorded. To identify morphological mutant’s growth abnormalities from the seedling stage until fruit harvesting were recorded. In addition, the frequency and segregation ratio of the mutant plants out of the total number of individuals in the same M_2_ family was calculated.

##### Photosynthetic pigment analysis

Chlorophyll a and b and carotenoids were extracted from 0.2 g of fresh leaves (obtained from the upper third of the leaf) of yellow green mutants and WT plants at the age of 100 DAS using ice-cold acetone. Extracts were centrifuged at 15000 rpm for 10 min at 10°C. The supernatant volume was diluted with 80% acetone. The chlorophyll contents were quantified spectrophotometrically following the method of (Porra et al., 1989).

##### Leaf anatomy

In the M_2_ generation, at 100 DAS, specimens were taken from the lower third of the leaf blade. The upper third of the leaves from the mutant and WT plants were taken for anatomical measurements. These specimens (1 cm long) were killed and fixed for 72 h in FAA (formalin, acetic acid, and ethanol) that was made using the following formula: 50 mL ethyl alcohol (95%), 5 mL glacial acetic acid, 10 mL formaldehyde (37–40%), and 35 mL distilled water. The selected materials were stained with both safranin and light green, and cleared in xylene (Berlyn and Miksche, 1976). The stained specimens were then washed in 50% ethyl alcohol, dehydrated in a normal butyl alcohol series, and embedded in paraffin wax with a melting point of 52–54°C. Sections were prepared using a rotary microtome at 12 microns, and selected sections were examined and photographed using a system Microscope (BX53). The dimensions of leaf blade sections were measured using the same system Microscope.

##### Statistical analysis

All obtained data from the M_1_ and M2 generations were subjected to analysis of variance (ANOVA) using SPSS software (Corp, 2010). The analyzed data were presented as means ± SE of two replicates in all measured parameters except for chlorophyll, which was done using three replicates.

## Results

### M_1_ Generation Observations

Out of the total number of seeds, 1009 seeds germinated (50.4%) showing 49.6% lethality during the seedling stage. After transplantation, further mortality occurred, with 939 plants (93.06%) reaching maturity (**Table 1**).

**Table 1:** The germination and survival percentage during the first generation (M_1_).

Treatments	Total No of seeds	No. of germinated seeds	Seed germination (%)	No. of survived seedlings	Survival (%) out of germinated seeds
EMS population	2000	1009	50.40	939.0	93.06
Wild type (WT)	50.00	47.00	94.00	47.00	100.0

As shown in **Table 2**, no significant change was observed in plant height in 80.05% of the M_1_ population as compared to the WT population, while 0.21% of the M_1_ plants were dwarf plants, 9.01% were short plants, 10.52% were long plants, and 0.21% were very long plants. The plant height in 68.08% of WT plants was 41–80 cm, 29.79% were 20–40 cm, and 2.12% of plants were 81–100 cm. The time to flowering in 0.97% of the mutant population was earlier than 70 DAS, 19.31% of plants flowered at 70–80 DAS, 50% flowered at 81–100 DAS, 28.65% flowered at 101–120 DAS, and 1.07% of plants flowered very late (after 120 DAS). On the other hand, in the WT population the flowering time was limited to just three categories: before 70 DAS, 70–80 DAS and 81–100 DAS, and the distribution of the WT plants in these categories was 12.77, 31.91, and 55.32%, respectively (**Table 2**).

**Table 2:** Categories of the growth pattern changes observed in the M_1_ generation as compared to the wild type (WT) population.

Categories	M_1_ population		WT population	
	Number of plants ± SE	%	Number of plants ± SE	%
**Plant height (cm)**				
Dwarf (<20 cm)	01.00 **± 01.00**	00.21	00.00 **± 00.00**	00.00
Short (20–40 cm)	42.00 **± 03.00**	09.01	07.00 **± 02.00**	29.79
Normal (41–80 cm)	373.0 **± 75.00**	80.05	16.00 **± 02.00**	68.08
Long (81–100 cm)	49.00 **± 08.00**	10.52	00.50 **± 00.50**	02.12
Very long (>100 cm)	01.00 **± 00.00**	00.21	00.00 **± 00.00**	00.00
**Number of days to flowering**				
Very early (<70 DAS)	04.50 **± 00.50**	00.97	03.00 **± 01.00**	12.77
Early (70–80 DAS)	90.00 **± 13.00**	19.31	07.50 **± 01.50**	31.91
Normal (81–100 DAS)	233.0 **± 13.00**	50.00	13.00 **± 02.00**	55.32
Late (101–120 DAS)	133.5 **± 07.50**	28.65	00.00 **± 00.00**	00.00
Very late (>120 DAS)	05.00 **± 01.00**	01.07	00.00 **± 00.00**	00.00
**Number of seeds/first fruit**				
Number of seeds (0)	20.50 **± 01.50**	04.40	00.00 **± 00.00**	00.00
Lower number (<50)	32.00 **± 09.00**	06.87	02.00 **± 00.00**	08.51
Normal (50–199)	407.0 **± 17.00**	87.34	21.50 **± 00.50**	91.48
High number (200–300)	05.00 **± 01.00**	01.07	00.00 **± 00.00**	00.00
Very much (>300)	01.50 **± 00.50**	00.32	00.00 **± 00.00**	00.00
**Leaf architecture**				
Aberrant	1	00.21	0	0

*DAS, days after sowing; all measurements for the mutant population were calculated as the average of 466 surviving plants; for the WT population all measurements were based on the average of 23.5 surviving plants (average of two replicates). The bolded values are the standard error of the means.*

**FIGURE 1:**
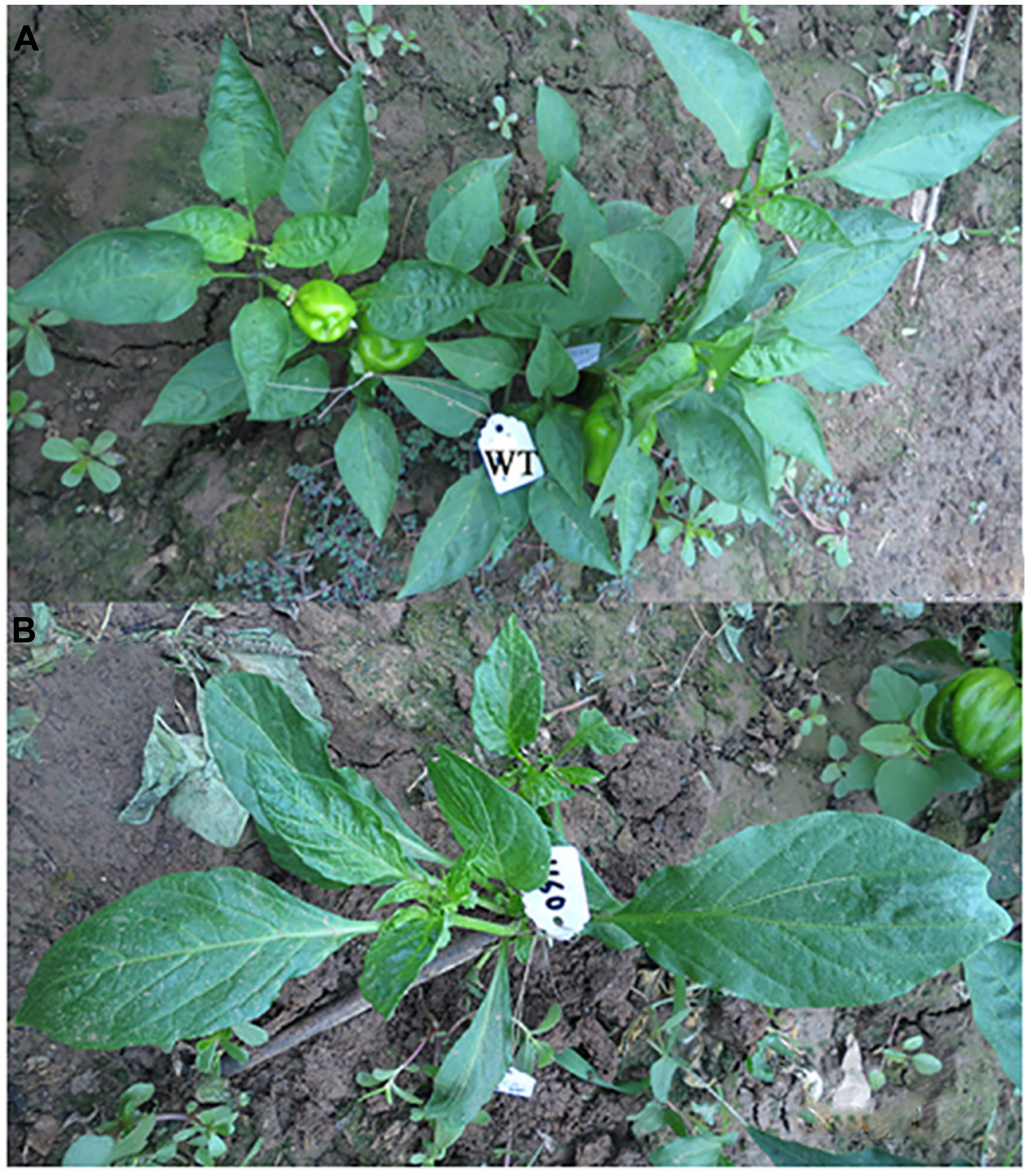
**Abnormal plant (leaf architecture and growth habit) observed during the first (M_1_) generation as compared to the wild type (WT)**. **(A)** WT plant, **(B)** mutant plant.

Regarding the number of seeds per first fruit, 4.4% of plants did not produce any seeds in the first fruit, 6.87% of plants had less than 50 seeds in first fruit, 87.34% had 50–199 seeds, 1.07% of plants have 200–300 seeds, and 0.32% of plants contained more than 300 seeds in the first fruit (**Table 2**). In the WT plants 91.48% of plants contained 50–199 seeds in the first fruit and 8.51% contained less than 50 seeds per first fruit. It was also observed during the M_1_ generation that there was one abnormal plant that had different leaf architecture (**Figure 1**; **Table 2**). In almost all of the studied characters, the growth performance significantly varied in the M_1_ generation compared to the WT population, which establishes a reasonable ground for EMS efficiency.

### M_2_ Generation Observations

#### Germination and Survival Percentages in M_2_ Generation

**FIGURE 2:**
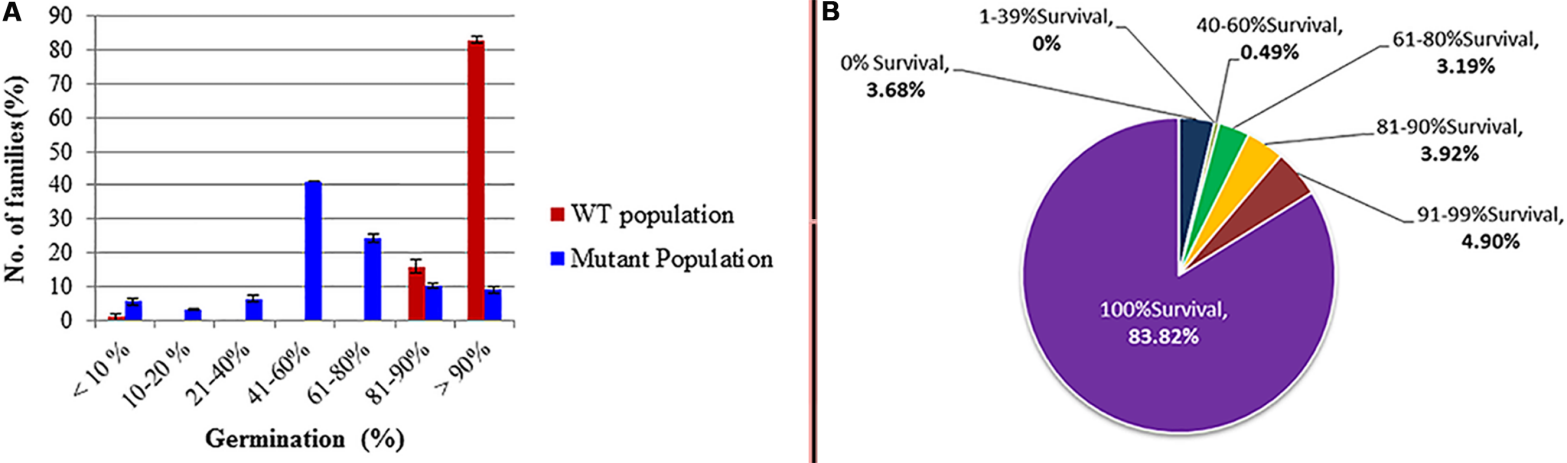
**Germination and survival percentages as affected by the ethyl methanesulphonate (EMS) treatment in the M_2_ generation wild type. (A)** Germination ratio; **(B)** survival ratio: it calculated out of the total number of germinated seeds (in the diagram the upper values indicates survival percentage categories and the lower value **(bold)** indicates the percent of survived families out of the total number of germinated families.

Data analysis of seed germination during the M_2_ generation (**Figure 2A**) showed that the effect of the mutagen on seed germination did not cease in the M_1_ but continued to affect the M_2_ generation. Further reduction was observed in the percentage of germinated seeds in different mutant families as compared to the WT. The germination ratio ranged from 41 to 60% in more than 40% of M_2_ families. Moreover, about 25% of families showed 61 to 80% germination, while the germination percentage in the WT families was about 80%. The survival ratios in the mutagenized and WT populations were recorded as the number of surviving seedlings out of the germinated seeds in all families (**Figure 2B**). About 83% of M_2_ families produced 100% viable seedlings, while 3.68% of families produced no viable seedlings, and the seedlings from other families showed survival ratio between 40–99%. On the other hand, the survival ratio in the WT population was 100%.

#### Screening of Chlorophyll Mutations During the Seedling Stage in M_2_ Generation

**FIGURE 3:**
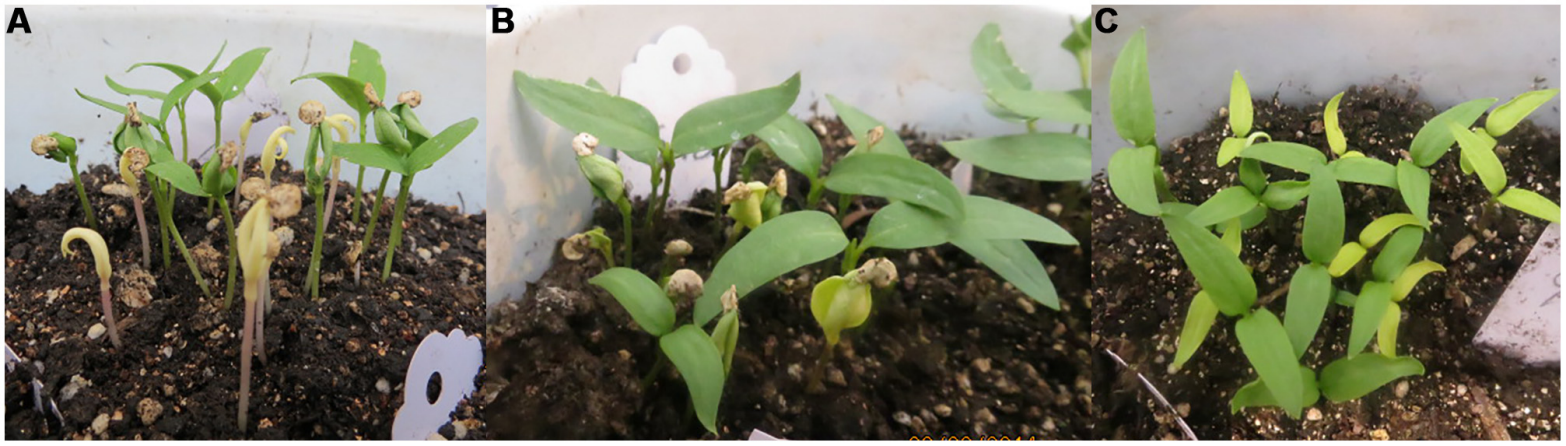
**Different types of chlorophyll mutants during the seedling stage in the M_2_ generation. (A)** Albino seedlings (totally white), **(B)** yellow seedlings, **(C)** pale green seedlings.

The appearance of chlorophyll mutants during seedling stage was a good indicator for EMS efficiency. As shown in **Table 3**, it was observed that among 500 families there were about 14 families had chlorophyll mutants, i.e., Albino, yellow, pale green seedlings (**Figure 3**). Furthermore, all the albino seedlings (totally white) were died, while some of yellow or pale green seedlings were able to survive till maturity stage.

**Table 3:** Ethyl methanesulphonate (EMS) induced chlorophyll mutations during seedling stage of the M_**2**_ generation.

Family No.	Total No. of plants in the family	Albino seedlings		Yellow seedlings		Pale green seedlings		Green (normal) seedlings	
		No.	%	No.	%	No.	%	No.	%
602	20.00	4.00	20.00	0.00	00.00	0.00	0.00	16	80.00
963	30.00	0.00	0.00	7.00	23.33	9.00	30.00	14	46.67
574	30.00	7.00	23.33	0.00	00.00	0.00	0.00	23	76.67
711	30.00	2.00	06.67	1.00	03.33	0.00	0.00	27	90.00
608	30.00	8.00	26.67	0.00	00.00	0.00	0.00	22	73.33
576	30.00	0.00	00.00	3.00	10.00	0.00	0.00	27	90.00
765	30.00	0.00	00.00	2.00	06.67	0.00	0.00	28	93.33
911	35.00	0.00	00.00	1.00	02.86	0.00	0.00	34	97.14
827	30.00	0.00	00.00	1.00	03.33	0.00	0.00	29	96.67
616	35.00	1.00	02.86	0.00	00.00	0.00	0.00	34	97.14
702	30.00	0.00	00.00	2.00	06.67	0.00	0.00	28	93.33
779	40.00	0.00	00.00	1.00	02.50	0.00	0.00	39	97.50
768	40.00	0.00	00.00	2.00	05.00	0.00	0.00	38	95.00
890	20.00	0.00	00.00	3.00	15.00	0.00	0.00	17	85.00

#### Characterization of Specific M_2_ Mutants

**FIGURE 4:**
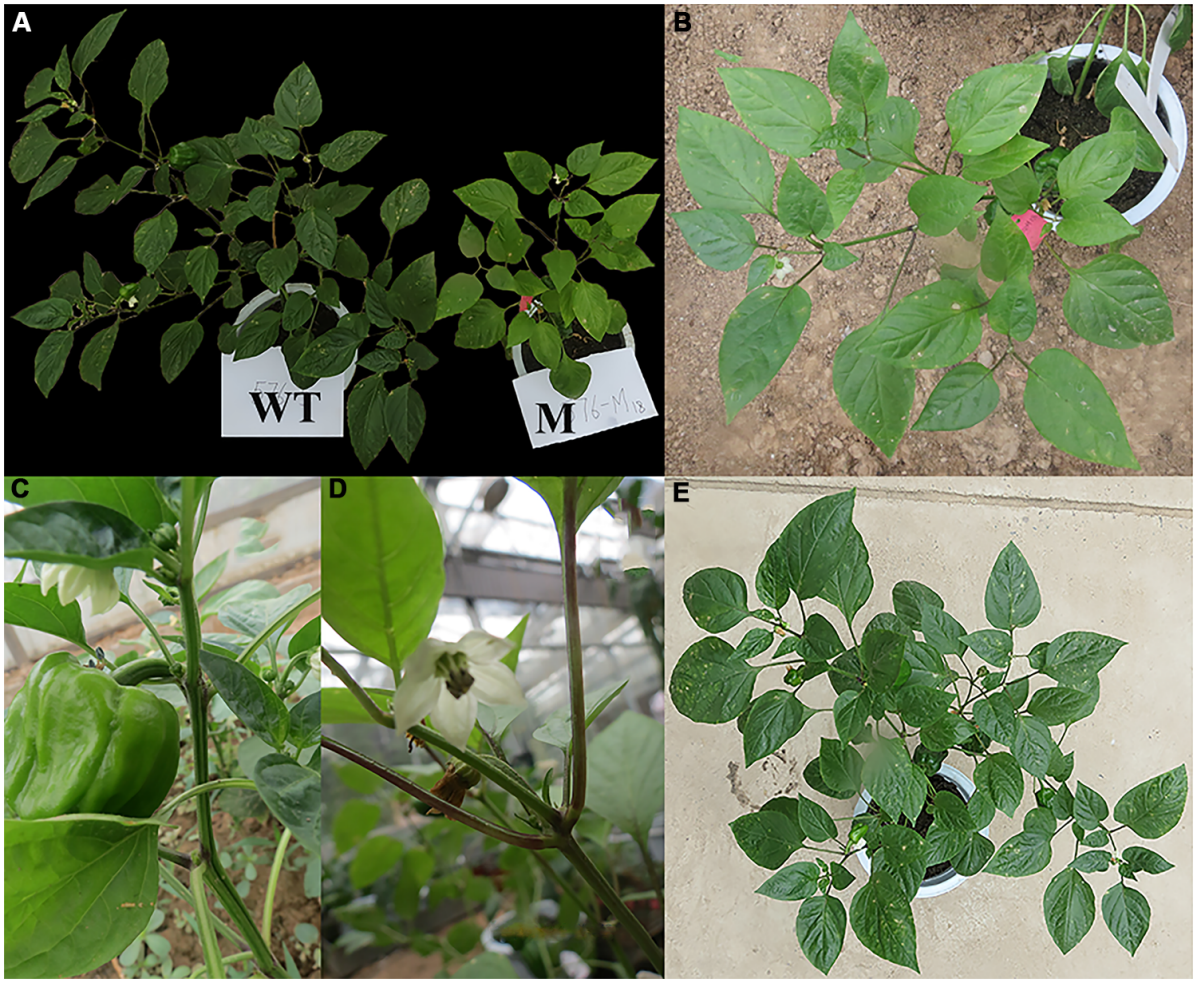
**Leaf color changes in the M_2_ generation as compared to WT plants. (A)** The mutant plant as compared to the WT, **(B)** the mutant plant, **(C)** near view for petiole and blade of the wild type leaf, **(D)** near view for the petiole (purple color) and blade (pale green color) of the mutant plant leaf, and **(E)** WT plant; WT, Wild type, M, Mutant.

**FIGURE 5:**
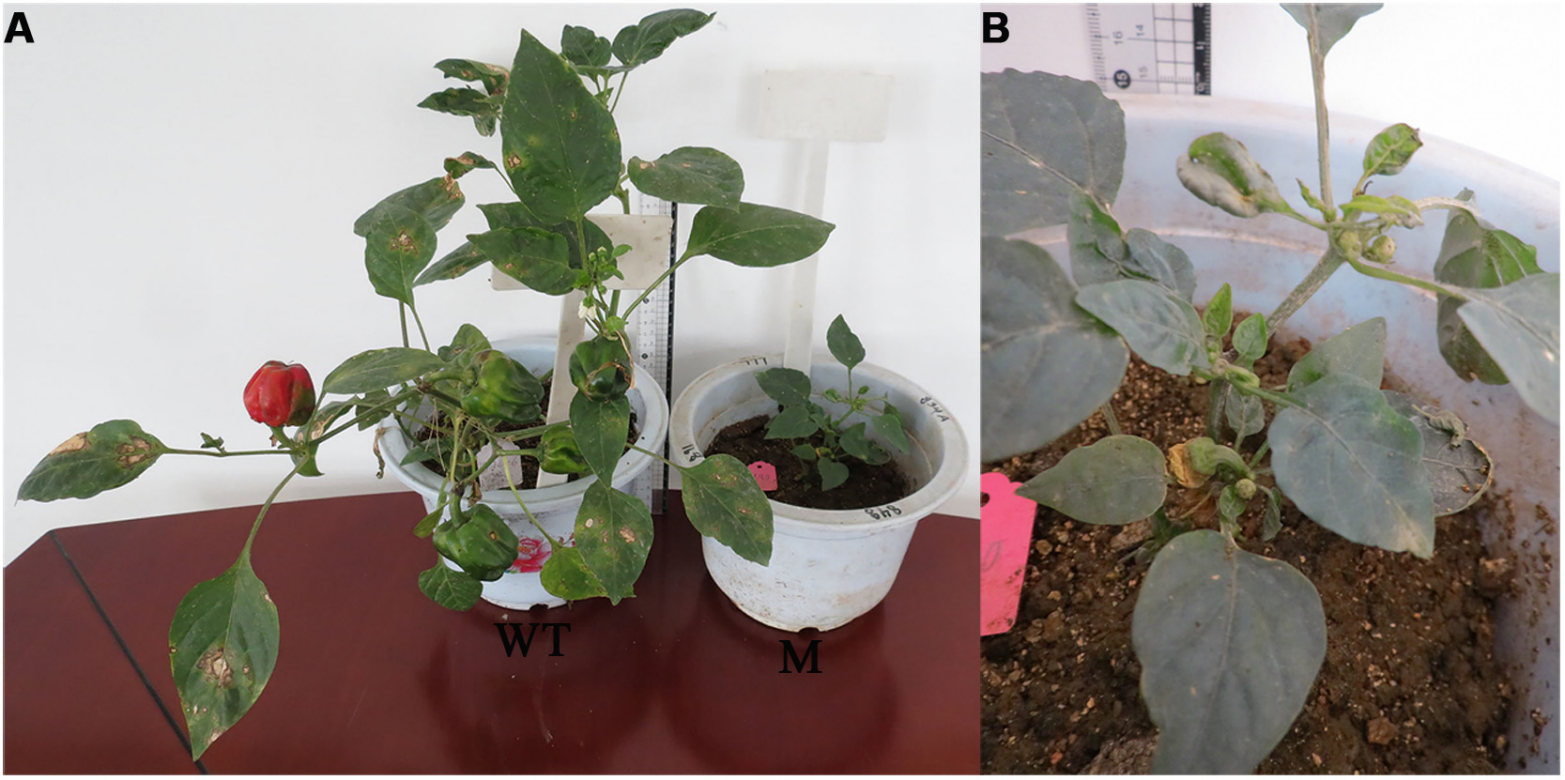
**Dwarf plant as compared to the WT plants during the M_2_ generation. (A)** WT plant (WT) and dwarf plant (M), **(B)** near view for the mutant (dwarf plant).

**FIGURE 6:**
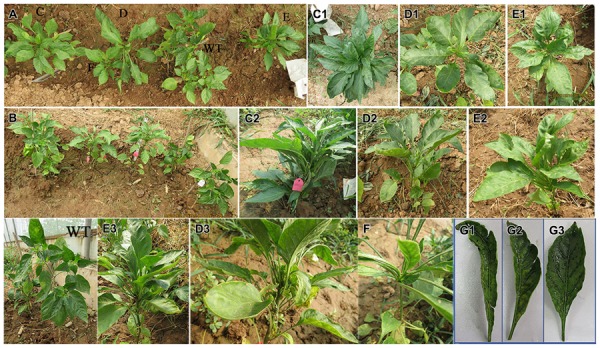
**Leaf architecture and growth habit mutation as compared to the WT in the M_2_ generation. (A)** Photo for all the mutant plants as compared to the WT, **(B–E)** mutant plants, **(C1–E1)** WT plants, **(C2–E2)** horizontal view for the mutant plants, **(G1–G3, E3, D3, F)** lateral view for the mutant plants.

The phenotypic mutations were identified by visual evaluation during the M_2_ generation. Among 500 families (15000 plants), three types of phenotypic mutations, e.g., leaf color, dwarf plants, leaf architecture, and growth habit, were clearly observed in three different families (**Figures 4–6**).

##### Leaf color mutation

During the M_2_ generation, a single family (No. 576) produced a distinct mutant phenotype with yellow leaves in one plant (No. 576–18). In this family there were three yellow plants out of the total number of plants in this family (30 plants) with a 10% frequency and segregation ratio of 1:10, which is not consistent with the classic Mendelian model (**Tables 3**–**5**). At the seedling stage, the leaf color of these mutant plants was yellow, which was very clear when compared to WT plants (**Figure 3B**). One plant of the three mutants survived until maturity, while the other two plants died. At maturity, the surviving plant also exhibited differences in leaf color, distinguishing it from the WT (**Figure 4**). In addition, the petioles of this mutant plant were dark purple, while the petioles of WT leaves were green (**Figures 4C,D**).

**Table 4:** Observed mutations during the second generation; its frequency (%) and segregation ratio.

Observed mutations	Total No. of plants/family	No. of mutant plants	Mutants’ frequency (%)	Segregation ratio
Growth habit and leaf shape mutation	17	4	23.52	4:1
Dwarf mutation	23	1	04.34	23:1
Leaf color mutation	30	3	10.00	10:1

**Table 5:** Phenotypic characters for the observed mutations as compared to the WT in the M_**2**_ generation.

Mutant No.	Plant height (cm)	No. of leaves/plant	Leaves color	No. of nodes/plant (main stem)	No. of flowers/plant	No. of fruits/plant	Final fruit color	No. seeds/first fruit
WT	45.0	84	Normal	15	26	3	Red	65
**Leaf architecture and growth habit mutation**								
891-1	30.0	58	Normal	5	0	0	–	–
891-2	13.5	19	Normal	7	0	0	–	–
891-3	19.0	16	Normal	5	0	0	–	–
891-4	14.0	42	Normal	9	1	1	–	–
**Dwarf mutation**								
690-1	06.0	7	Normal	4	7	0	–	–
**Leaf colored mutation**								
576-18	29.0	30	Yellow	7	4	2	Bright red	10
576-5	59.0	62	Dark green	15	15	3	Red	18

*Numbers 891, 690, and 576 indicate the family number during the second generation and the second number (1, 2, 3, 4, 5, and 18) after the family number indicates the mutant plant number in the family.*

The chlorophyll content of leaves in all plants of this family as well as WT plants were determined to characterize the nature of the yellow leaf phenotype (**Table 6**). The results indicated that the leaves of one plant (No. 576-18) from the family contained 62.19% less chlorophyll a and 64.06% less chlorophyll b than those from the WT. The growth parameters recorded for this yellow plant included: plant height (29 cm), number of leaves (30 leaves), number of internodes (seven), number of flowers (four), number of fruits (two), and number of seeds per first fruit (10), while the WT plants had: 45, 84, 15, 26, 3, and 65 for the above-mentioned traits, respectively (**Table 5**).

**Table 6:** Chlorophyll a and b and carotenoids (mg/g FW) in the assayed leaf colored mutants as compared to the WT in the M_**2**_ generation.

Plant No.	Chlorophyll A ± SE	Chlorophyll B ± SE	Carotenoids ± SE	Total Chlorophyll ± SE	Chlorophyll A/B ± SE
WT	1.64 ± 0.009	0.64 ± 0.009	0.87 ± 0.011	2.27 ± 0.017	2.57 ± 0.029
576-18	1.02 ± 0.048	0.41 ± 0.008	0.77 ± 0.042	1.43 ± 0.036	2.48 ± 0.027

*SE, standard error; FW, fresh weight.*

##### Dwarf mutation

The dwarf mutant that was isolated was categorized as less than 10 cm in height with short internodes and compact nodes. Among the total numbers of plants in this family (30 plants) 23 plants survived and one of them was a dwarf mutant (No. 690-1). The dwarf mutant had a frequency of 4.34% and a segregation ratio 23:1, which was not consistent with the classic Mendelian model as shown in **Tables 4** and **5**. At 100 DAS, this dwarf plant had thick, rough and fibrous leaves, and a ribbed, rough, and thick stem, while the WT plants had smooth green leaves and stems (**Figure 5**). The mutant achieved a height of 6 cm and produced seven sterile flowers as compared to the WT, which had an average height of 45 cm and 26 fertile flowers.

##### Leaf architecture and growth habit mutation

**FIGURE 7:**
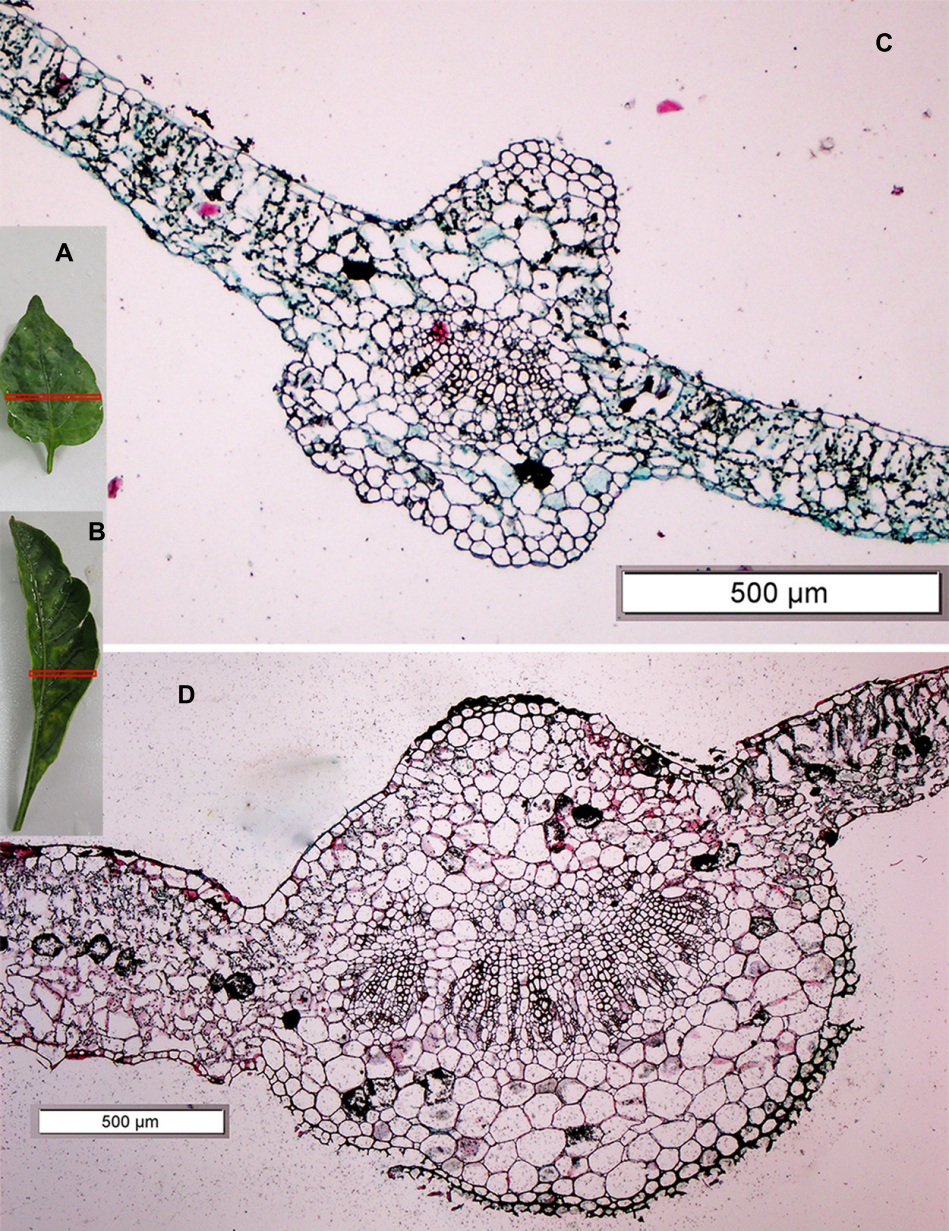
**A leaf cross section of the leaf architecture and altered growth habit mutant plant as compared to the WT. (A,B)** The third leaf of the WT plant and mutant plant, respectively, Red rectangle on the leaves (A,B) shows the position of cross section where taken, **(C,D)** the cross section under microscope of the WT plant and mutant plant, respectively.

Four mutants with an altered growth habit were identified in the M_2_ population (**Figure 6**), although, these mutations did not appear in M_1_ generation. These four mutant plants were found in one family (No. 891) and had a frequency of 23.52% and a segregation ratio 4:1, which is consistent with the classic Mendelian model as shown in **Table 4**. Leaf blades of these four mutant plants were thicker and longer than those of the WT (**Figure 7**). In addition, all of these mutant plants lacked branches during their growth stages.

The growth habit of the four mutant plants was different when compared with the WT. In the WT B12 cultivar, growth was determinate; the shoot apical meristem produced approximately eight true leaves on the main stem and then terminated in a flower. Further growth continued from the uppermost axillary meristems creating branches and so on. The observed mutations caused a dramatic change in plant architecture. In one mutant plant (No. 891-1), many leaves were produced from the third node and then growth continued by producing one leaf for each node until the sixth node, where many leaves were produced at the same node (sixth) without branching (**Figures 6C1,C2**). In the other three mutants (No. 891-2, 891-3, and 891-4), plants started to produce leaves at the fifth node without branching. Furthermore, one of the mutant plants (No. 891-4) induced one lateral fruit at the second node (**Figures 6E1,E2**). The results shown in **Table 5** indicated that the growth habit of all mutant plants (plant height, number of leaves, and number of nodes) were different than those of the WT plants.

Comparing the third leaf cross section of the mutant and WT using a scanning microscope (**Figure 7**), we found that the right and left sides of the leaf blade in the mutant plant were very thick (551.55 and 530.00 μm, respectively) as compared to the WT (139.24 and 211.55 μm, respectively). In addition, the leaf blade of the mutant contained thicker palisade and spongy tissue than the WT. A cross section of the main midrib also showed clear differences in the xylem and collenchyma tissue, as it was very thick in the mutant plant compared to the WT. Xylem and collenchyma tissue thickness in the mutant plant was 242.42 and 530.34 μm, while in the WT leaf it was 61.85 and 240.53 μm, respectively (**Table 7**). These results indicated that the leaves of the mutant plants are thicker than the WT (**Figure 7**).

**Table 7:** Leaf sections of the mutant and WT plants using a scanning microscope.

Parameters	Thickness (μm)	
	Mutant	WT
Leaf blade (right side)	0551.55	139.24
Leaf blade (left side)	0530.00	211.55
Midrib	1474.57	639.96
Xylem tissues	0242.42	061.85
Collenchyma tissues	0530.34	240.53

## Discussion

In mutation breeding programs, the selection of an effective and efficient mutagen concentration and growth condition is essential to produce a high frequency of desirable mutations (Wani, 2009; Arisha et al., 2014). Arisha et al. (2014) studied the LD_50_ in the same pepper cultivar and found that 0.6% V/V EMS was the concentration that produced about 50% lethality. The reduction in germination may be due to the seeds absorbing the mutagen, which subsequently reaches the meristemic region and affects the germ cell (Serrat et al., 2014). Also, a reduction in germination may be because of the damage of cell constituents (Kumar et al., 2013), alteration of enzyme activity or delay or inhibition of physiological and biological processes (Talebi et al., 2012).

In the present investigation the growth behaviors (plant height, number of days to flowering, and number of seeds per first fruit) of the mutant population exhibited changes in the above-mentioned characters as compared to the WT population. This might be due to the biological damage of the embryo as induced by EMS, and this in turn reflected on the plant growth behavior until maturity (Arisha et al., 2014). Genetically, during the M_1_ generation the probability of the occurrence of phenotypic mutation is extremely low and only dominant mutations can be identified (Roychowdhury and Tah, 2013). In the current study, during the M_1_ generation, among 2000 mutagenized seeds, only one phenotypic mutation was observed. This mutant had different leaf shapes and a different growth habit (**Figure 1**). In concordance with the current results, Honda et al. (2006) surveyed mutagenized pepper plants for mutants during the M_1_ generation and found two dwarf plants and one plant with a yellow fruit pericarp.

The harmful effects of EMS did not stop at the M_1_ generation, but also affected the M_2_ generation. In the M_2_ generation, data analysis showed a significant reduction in seed germination in most families as compared to the WT population. The survival ratio was slightly affected and about 83% of the mutant population survived until maturity, which was similar to the rate of the WT population. Furthermore, during the M_2_ generation a chlorophyll defect appeared. It was found that EMS reduced seed germination and survival ratio during the M_1_ generation and that effect continued in the second generation in peppers (Alcantara et al., 1996; Jabeen and Mirza, 2002), tomatoes (Saba and Mirza, 2002), and *Dianthus* (Roychowdhury et al., 2012). This might be due to the effect of EMS on M_0_ seeds, which negatively affected the embryo, germination and this negative effect reflected on the plant growth and fruit formation of M_1_ plants (including fruits and formed seeds inside the fruit), which in turn produced bad quality of seeds (Abdul-Salam, 2007). In agreement with our results, previous studies found that the changes in the M_2_ generation appeared during the seedling stage in the formation of chlorophyll or slow growth and also affected the growth until maturity (Pawar et al., 2010).

During the M_2_ generation, the chance for identifying visible changes or phenotypic mutations should be higher and mostly due to genetics. Therefore observed mutations in the M_2_ generation are considered more stable (Parry et al., 2009). In the present work, during the M_2_ generation, among 500 families, there were three types of mutations in three families that could be visibly confirmed. One type of visible mutation was observed in one plant that had a uniform yellow–green color, characterizing chlorophyll deficiency. The plant with this mutation was able to survive until maturity. In agreement with our results, previous investigations found that a significant change in chlorophyll always resulted in the variation of leaf color (Hooda et al., 2004; Chen et al., 2007; Pawar et al., 2010). Previous studies reported that chlorophyll development seems to be controlled by many genes that are located on different chromosomes (Larkin and Scowcroft, 1981; Wang et al., 2013). Mutations affecting the production of chlorophyll are important for identifying gene function and the elucidation of chlorophyll metabolism and its regulation (Wu et al., 2007). Identifying a gene that regulates leaf color can be considered a critical issue because it can offer an alternative color to pepper breeders. Therefore, we are currently conducting further molecular analyses to detect the changes in genes that are responsible for chlorina in mutant peppers.

Dwarf mutants in peppers are crucial to understanding the regulatory mechanisms for plant growth and development (Ashikari et al., 1999). Furthermore, dwarfism is a favorable character for breeding programs. In the present investigation, the second type of mutation observed was dwarfism, and we recorded a plant with a height less than 10 cm with short internodes and thick, rough, fibrous, green leaves. Dwarf mutants were also reported earlier in peppers (Lippert et al., 1964; Alcantara et al., 1996; Jabeen and Mirza, 2004), which might be due to the inhibition of the elongation of epidermal cells, or a defect in GA biosynthesis (Fridborg et al., 1999). Discovery of recessive, monogenic mutations causing dwarfism is very important to allow for easier identification of the genes that control pepper growth (Wang and Bosland, 2006).

Another mutation that we observed affected the plant architecture and leaf texture. In plants with this mutation, the changes appeared in more than one character. In support of our results, mutations affecting branching pattern were previously observed in peppers (Mao et al., 2000). The mutant identified in the present study could be considered promising for establishing a reasonable ground for the genes controlling pepper architecture. The multiple phenotypes could be caused by one mutation with pleiotropic effects, or multiple mutations. The genetic control of plant architecture has been studied mostly in limited model plants such as *arabidopsis*, rice, maize, tomato, and petunia (Izawa, 2007; Wang and Li, 2008; Castel et al., 2010). In addition, these mutants also produced very thick leaves as compared to the WT.

Finally, we observed that two types of detected mutations in the present study are not consistent with the classic Mendelian model. The unusual segregation ratio in the dwarf (10:1) and leaf color mutants (23:1) might be due to the fact that changed characters are controlled by more than one gene and different genes interact to give this observed mutation (Hartl and Clark, 1997). In addition, unusual segregation ratios may also be due to using a fewer number of individuals in the mutant population, which may not have been enough to result in the usual segregation ratios (Hartl and Clark, 1997). Therefore, we are currently conducting further molecular study on this observed mutation to detect the genes responsible for inducing these changes, which can ultimately be used to improve the B12 pepper cultivar.

## Conclusion

In the present study, EMS was used to induce genetic variability in the B12 cultivar of *C. annuum*. During the M_1_ generation there was a clear difference between the mutant and the WT populations. In the M_1_ we observed one visible mutation in which the plant had a change in leaf shape and growth habit. During the M_2_ generation a chlorophyll defect was observed at the seedling stage. Three phenotypic mutations were recognized during the M_2_ generation: yellow leaf color, dwarf stature, and a mutation in plant architecture and leaf shape. The yellow leaf color mutation can be used to understand the gene(s) that regulate chlorophyll metabolism and function. The dwarf mutation may allow for identification of the genes controlling pepper growth, which is a commercially desirable trait, as dwarf plants are easy to maintain and can grow in limited space. The identified mutants in plant architecture and leaf shape can provide an understanding of the genes controlling pepper architecture. Further work is needed to analyze these mutants and determine the genetic reasons underlying the visible changes in order to genetically improve *C. annuum* cultivars.

## Conflict of Interest Statement

The authors declare that the research was conducted in the absence of any commercial or financial relationships that could be construed as a potential conflict of interest.

## Abbreviations

DAS: days after sowing
EMS: ethyl methanesulphonate
GA: Gibberellin.
