# Supplementary figures and images for: Ethyl methane sulfonate induced mutations in M2 generation and physiological variations in M1 generation of peppers (Capsicum annuum L.)
Source: Front Plant Sci. 2015 Jun 4;6:399. doi: 10.3389/fpls.2015.00399 (PMC4454883; doi:10.3389/fpls.2015.00399)

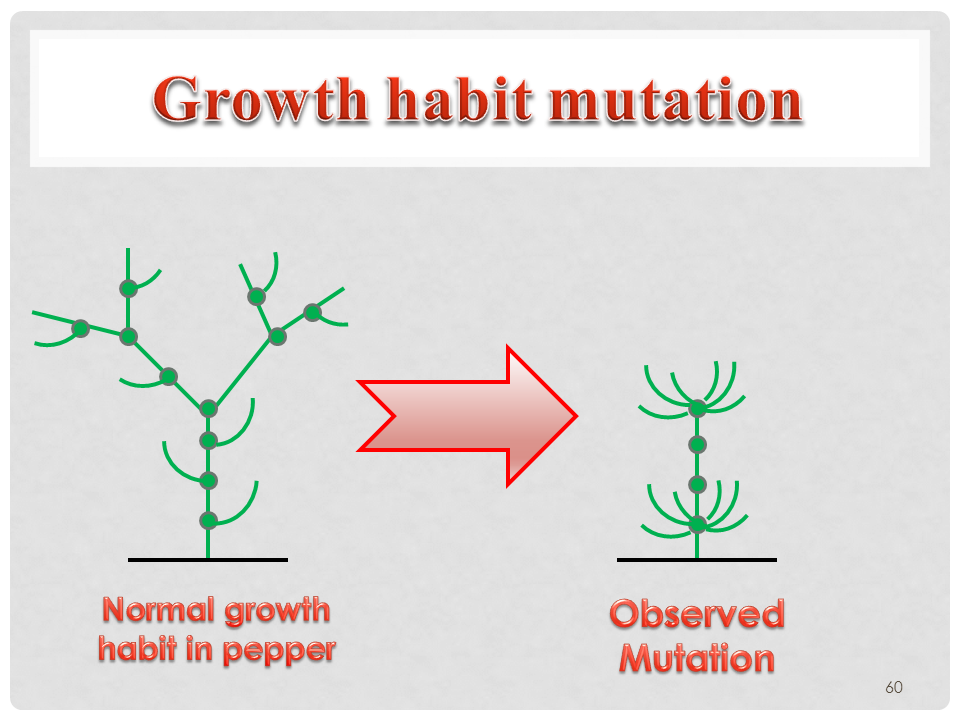

Supplement: Supplementary file 1 [file Image_1.TIF]
